# Splicing mutations in human genetic disorders: examples, detection, and confirmation

**DOI:** 10.1007/s13353-018-0444-7

**Published:** 2018-04-21

**Authors:** Abramowicz Anna, Gos Monika

**Affiliations:** Department of Medical Genetics, Institute of Mother and Child, Kasprzaka 17a, 01-211 Warsaw, Poland

**Keywords:** Splicing mutation, Spliceosome, Pre-mRNA splicing, Splicing enhancers and silencers

## Abstract

Precise pre-mRNA splicing, essential for appropriate protein translation, depends on the presence of consensus “cis” sequences that define exon-intron boundaries and regulatory sequences recognized by splicing machinery. Point mutations at these consensus sequences can cause improper exon and intron recognition and may result in the formation of an aberrant transcript of the mutated gene. The splicing mutation may occur in both introns and exons and disrupt existing splice sites or splicing regulatory sequences (intronic and exonic splicing silencers and enhancers), create new ones, or activate the cryptic ones. Usually such mutations result in errors during the splicing process and may lead to improper intron removal and thus cause alterations of the open reading frame. Recent research has underlined the abundance and importance of splicing mutations in the etiology of inherited diseases. The application of modern techniques allowed to identify synonymous and nonsynonymous variants as well as deep intronic mutations that affected pre-mRNA splicing. The bioinformatic algorithms can be applied as a tool to assess the possible effect of the identified changes. However, it should be underlined that the results of such tests are only predictive, and the exact effect of the specific mutation should be verified in functional studies. This article summarizes the current knowledge about the “splicing mutations” and methods that help to identify such changes in clinical diagnosis.

## Introduction

**Fig. 1:**
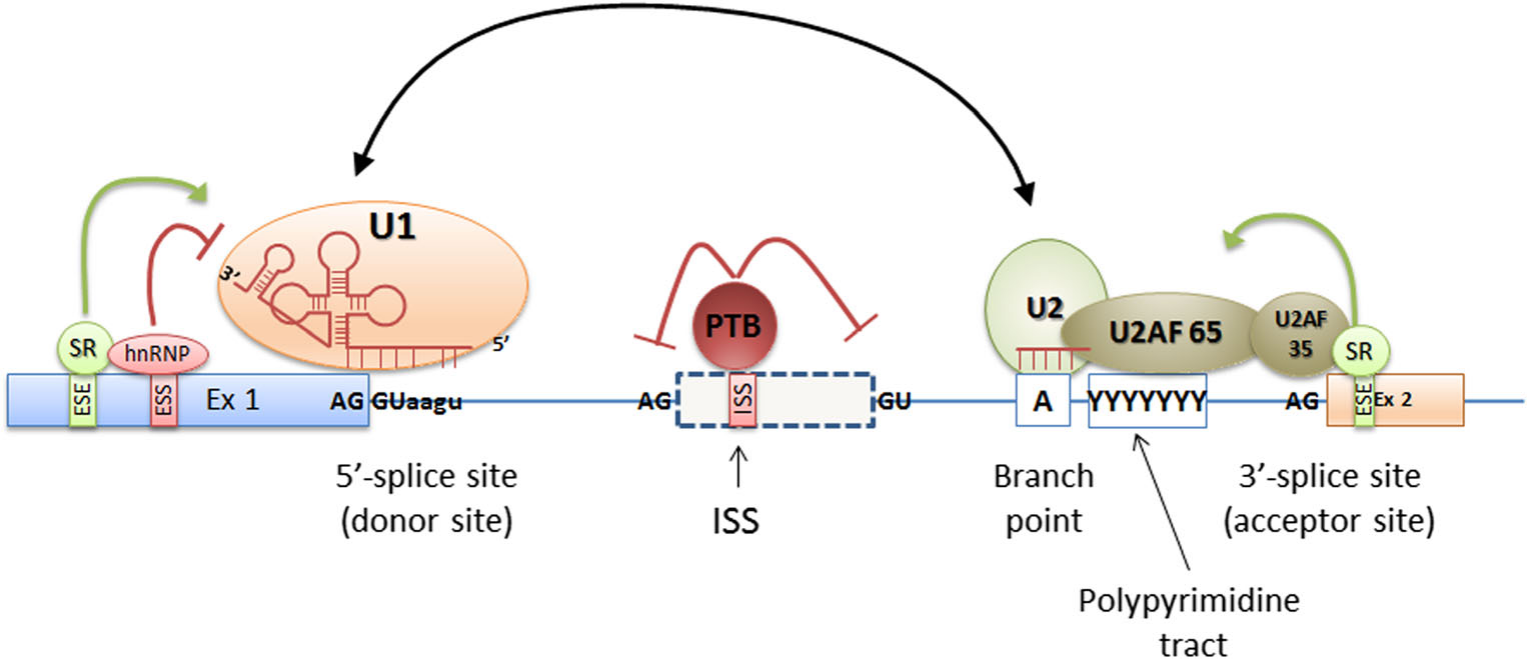
The schematic localization of the *cis* and *trans* splicing elements. The *cis* elements are the DNA sequences that include donor (5′) and acceptor (3′) splice sites, branch point and polypyrimidine tract sequences, and splicing silencers and enhancers. Donor and acceptor sites are evolutionary conserved and are usually defined by GT and AG nucleotides at the 5′ and 3′ ends of the intron, respectively. The branch site and the polypyrimidine tract sequences are highly degenerated and together with donor and acceptor sites are recognized by the elements of the splicing complex called spliceosome. Spliceosome proteins together with splicing repressors and activators recognize *cis* splicing elements and are called *trans-*acting elements

The mature mRNA contains only coding sequences—the intronic ones are removed from the transcript during the splicing process (Rahman et al. 2015). This process is held in nucleus and is dependent from the presence and interaction between the so-called *cis* and *trans* elements. The *cis* elements are the DNA sequences that define exons, introns, and other regulatory sequences necessary for proper splicing. They are termed consensus splice site sequences and include donor (5′) and acceptor (3′) splice sites, branch point and polypyrimidine tract sequences, and auxiliary *cis* elements including splicing silencers and enhancers (Fig. 1).

**Fig. 2:**
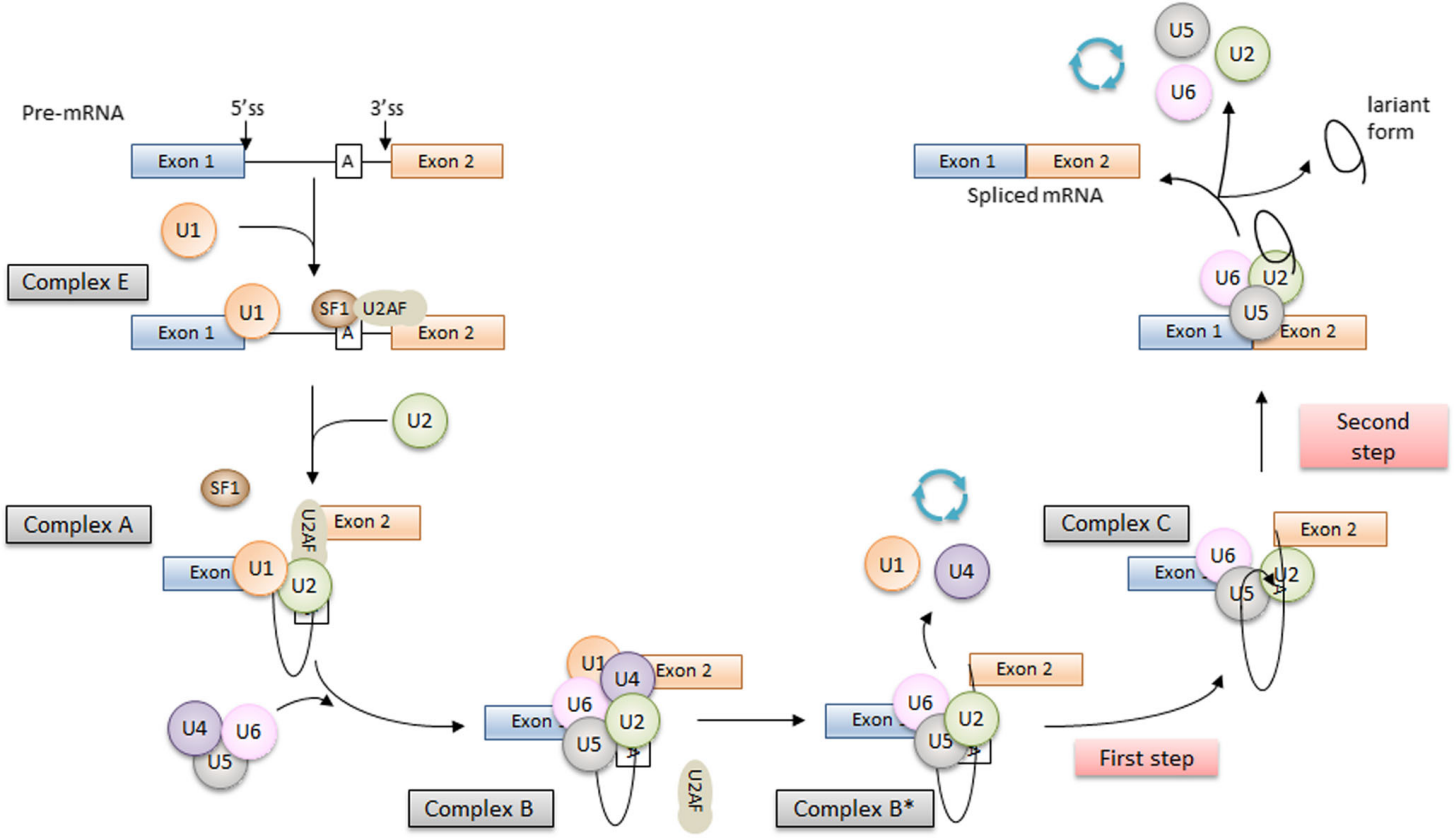
The scheme of the splicing process. The splicing process is performed in two steps. The first step is the recognition of the splicing sites at intron/exon junctions, and the second one is the intron removal and exon ends joining. During the splicing process, four complexes between the pre-mRNA and spliceosome are formed. The first one is the early complex (E). The U1 snRNP recognizes and binds to the complementary AG-GU sequence at the donor splice site (5′ end of the intron). In the same time, the SF1 protein binds to the branch point. The SF1 is recognized and bound by the U2AF65 protein that also binds to the polypyrimidine sequence located between the branch point and 3′ end of the intron. Then, the SF1 is displaced from the branch point by the U2 snRNP, and the ATP-dependent (A) complex is formed. The interaction between the branch point and the U2snRNP protein is stabilized by specific RNA helicases (Prp5 and Sub2), and this is a signal for the recruitment of U4/5/6 tri-snRNP and formation of the B complex (pre-catalytic spliceosome). Further action of additional RNA helicases leads to change of spliceosome conformation that leads to the release of U1 and U4 snRNPs, the interaction between U6 with U2 snRNP, and the formation of a pre-mRNA loop and the C complex. In this complex, two transesterification reactions take place, intron is removed, and ends of exon are joined (Fredericks et al. 2015; Tazi et al. 2009)

In brief, the splicing process is catalyzed by the spliceosome, a protein-RNA complex containing five small nuclear ribonucleoproteins (snRNPs, U1, U2, U4–U6) and over 300 different proteins. The presence of small nuclear RNAs (snRNAs) in snRNPs allows to form complementary RNA-RNA complexes and thus identify the specific sequences of the splicing sites by the spliceosome (Fig. 2, Faustino and Cooper 2003).

In most of cases (98.7%), the exon/intron boundary sequences contain GT and AG motifs at the 5′ and 3′ ends of the intron, respectively. Noncanonical GC-AG and AT-AC sequences at the splice sites occur in 0.56 and 0.09% of the splice site pairs. The noncanonical GC-AG splice site, like the GT-AG splice pair, is processed by major spliceosome complex. The AT-AC splicing motifs, that are present in U-12 type introns, are recognized by functionally relevant U12-dependent spliceosome called minor spliceosome (Parada et al. 2014). This complex is similar to the major spliceosome with the exception of several snRNPs. The U1, U2, U4, and U6 are replaced with U11, U12, U4atac, and U6atac, respectively. The U11/U12 proteins bind to their target sequences as a preformed di-snRNP complex (Turunen et al. 2013).

Any errors during the splicing process may lead to improper intron removal and thus cause alterations of the open reading frame. Therefore, the spliceosome complex has to correctly recognize and cut out the intronic sequences from the pre-mRNA molecule. The proper identification of the splice site is demanding as the consensus sequences are very short and there are many other sequences similar to the consensus motifs of the canonical splice sites. These sequences are known as cryptic, noncanonical, or pseudo splice site sequences. Sometimes, a pseudo splice site matches the consensus sequences better than the natural one, but because of the lack of *cis*-acting regulatory elements, necessary for the proper exon identification, it is not used in the splicing process (Cartegni et al. 2002). The *cis*-regulatory elements include exonic and intronic splicing enhancers (ESE and ISE, respectively) and exonic and intronic splicing silencers (ESS and ISS, respectively) (Glisovic et al. 2008). These elements are recognized by specific splicing repressors and activators (*trans-*acting elements) that help to properly carry out the splicing process.

Splicing enhancers are conserved nucleotide sequences, specifically recognized by the serine and arginine-rich proteins (SR proteins). These proteins bind to the specific intronic/exonic splicing enhancers via RNA recognition motifs and interact with other splicing factors such as snRNP proteins. This interaction is mediated by the C-terminal RS domain of the SR proteins. The SR proteins establish an RNA/RNA interaction at the 5′ splice site and the branch point, assist the formation of an early spliceosomal complex E, and seem to promote the inclusion of exons they are bound with (Caceres and Kornblihtt 2002; Lee and Rio 2015).

Also, negative regulators are involved in the regulation of the splicing process. These proteins, mainly heterogeneous nuclear ribonucleoproteins (hnRNP), bind to exonic and intronic splicing silencers (Wagner and Garcia-Blanco 2001). The hnRNP protein family includes different proteins with molecular weight between 34 and 120 kDa, called alphabetically from hnRNP A to hnRNP U (Han et al. 2010). The hnRNP proteins differ with RNA-binding domain (RBD) that is responsible for the interaction specificity (Geuens et al. 2016). Their regulatory action may involve the “looping-out” mechanism—the hnRNP proteins bind to both ends of the exon, and because of their interaction, the exon is “looped out” and becomes blocked to the spliceosome complex. Another proposed mechanism suggests that hnRNPs coat the exon and act as an antagonist of splicing enhancers thus preventing their binding (Caceres and Kornblihtt 2002; Zhang et al. 2008a, b; Kolovos et al. 2012).

To summarize, the splicing process is a complex event that is important for proper protein synthesis. Any alterations of this process might lead to the decrease of the level of the specific messenger RNA and thus deprivation of the protein level that can result in aberrant cellular metabolism and/or function (Chabot and Shkreta 2016). The aberrant splicing of pre-mRNA due to the presence of point mutations, e.g., nucleotide substitutions, that alter the consensus splicing regulatory sequences in a specific gene, may lead to the specific hereditary monogenic disorders. This article summarizes the current knowledge about the “splicing mutations” and methods that help to identify such changes in clinical diagnosis.

## *Cis*-element splicing mutations

**Fig. 3:**
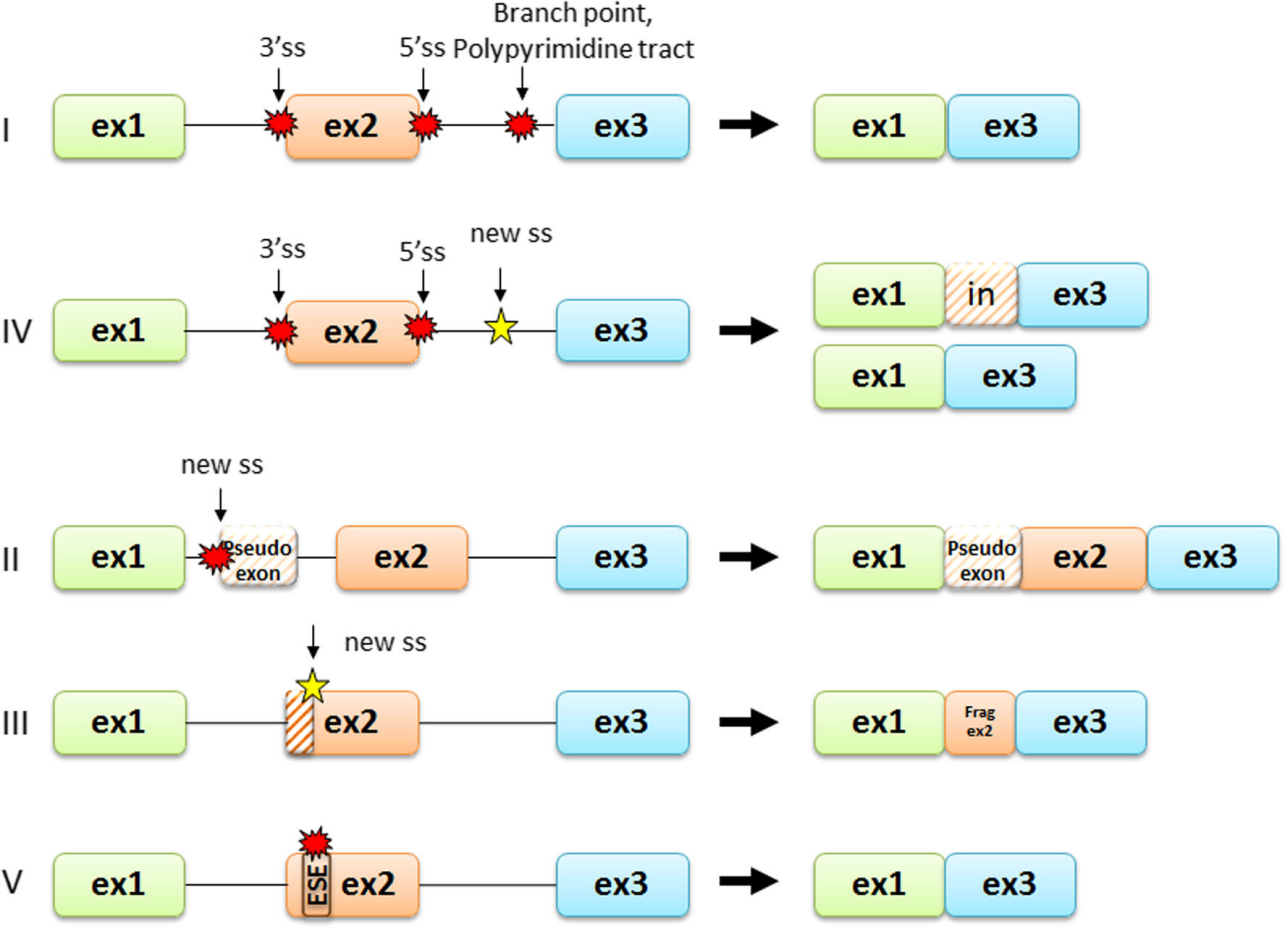
The types of splicing mutations. According to Wimmer et al. (2007), splicing mutations can be briefly divided into five categories: (1) splicing mutations within the canonical splice sites leading to whole exon skipping, (2) deep intronic variants creating new splice sites resulting in the inclusion of cryptic exons, (3) exonic single nucleotide variants creating new splice sites that result in the loss of an exon fragment, (4) variants in the canonical splice sites resulting in the usage of the cryptic exonic or intronic splice site that leads to the inclusion of an intron fragment or exon fragment skipping, and (5) mutations within the exon, usually leading to ESE disruption, resulting in the entire exon skipping

In general, the term splicing mutations usually refers to the point mutation at the *cis* consensus sequences that effects in improper exon and intron recognition in messenger RNA and results in the generation of an aberrant transcript of the mutated gene. The splicing mutation may occur in both introns and exons and disrupt existing splice sites, create new ones, or activate the cryptic ones. They also can influence splicing enhancers and silencers binding or alter the secondary structure of messenger RNA and therefore prevent the binding of the spliceosome elements. Usually such mutations result in exon/exon fragment skipping during the pre-mRNA splicing. If the resulting deletion is an in-frame one, the shorter protein will be synthetized. When the deletion results in the shift of the open reading frame, a premature stop codon (PTC) can be introduced and shorter protein can be produced. However, the presence of the PTC in the transcript usually leads to faster mRNA degradation during a protective process called nonsense mediated decay (NMD). The degradation of the defective messenger RNA prevents aberrant protein synthesis and has the same effect as gene deletion or nonsense mutation (Sterne-Weiler and Sanford 2014). According to Wimmer et al. (2007), splicing mutations can be briefly divided into five categories that are further briefly discussed (Fig. 3).

### Mutations in the canonical acceptor and donor sites (type I and IV mutations)

In general, mutations in the canonical acceptor and donor sites affect strongly conserved sequences that define exon-intron boundaries. The 5′ splice site (CAG/GUAAGU sequence) and 3′ splice site (NYAG/G sequence) are recognized by the elements of the spliceosome. Therefore, any variants in these canonical sequences might alter interaction between pre-mRNA and proteins involved in the intron removal. The most classical mutations affect + 1 and + 2 residues at the 5′ donor splice site and − 1 and − 2 residues at the 3′ acceptor splice site. The meta-analysis of splicing mutations (478 mutations in 38 selected genes) indicated that the donor splice site mutations were more prevalent than the acceptor splice site variants (ratio 1.5:1) (Krawczak et al. 2007). Similar results were obtained if a single gene was taken into consideration. Pros et al. (2008) had found that the splicing mutations in the *NF1* gene more frequently affected 5′ splice site (65%) than the 3′ one (35%). According to HGMD® Professional database (release 2017.3), this difference is lower and equals to 56% and 44% for 5′ and 3′ splice site, respectively.

Mutations at the canonical splice sequences usually lead to single exon skipping (type I mutation, see Table 1). However, if the splice site is weak and the presence of mutation uncovers the cryptic splice site in a neighboring exon or intron, this alternative site can be used in the splicing process (type IV mutation). This can lead to the inclusion of the intron fragment or the removal of an exon fragment if the cryptic splice site is present in intron or exon, respectively. In such situation, several distinct transcripts can be generated like in the case of the c.1525-1G>A variant in intron 9 of the *CFTR* gene. Detailed analysis revealed the presence of three different mRNA isoforms that utilized distinct alternative splice sites located within intron 10 and exon 10 at positions c.1610–1611 and c.1678–1679 and were lacking whole exon 10 or its fragments, respectively (Ramalho et al. 2003).

**Table 1:** Examples of different splicing mutations

Disease	Gene	Mutation	Type of splicing mutation	Effect on pre-mRNA splicing	Commentary	Reference
Type I						
Autosomal dominant congenital cataract	*MIP*	c.606+1G>A	Donor splice site mutation	Exon 3 skipping	Point mutation at the canonical splice site leads to the whole exon skipping	Zeng et al. (2013)
Neurofibromatosis type 1	*NF1*	c.1845+1G>A		Exon 15 and 16 skipping	Multi-exon deletion, mutation, and weak splicing sites force 2 exon skipping, description in the main text	Fang et al. (2001)
Ehlers-Danlos syndrome	*COL5A2*	c.925-2A>G	Acceptor splice site mutation	Several transcripts: (1) exon 7 and 6 skipping and (2) exon 7 skipping	Spliceosome utilizes the donor-splice site of exon 5 and the acceptor-splice site of exon 8 leads to skipping of both exons 6 and 7	Symoens et al. (2011)
Succinyl-CoA:3-ketoacid CoA transferase (SCOT) deficiency	*OXCT1*	c.1248+5G>A	Donor splice site mutation	Skipping of exons 12 and 13	Mutation leads to “splicing paralysis” and the removal of whole intron 11-mutated intron 13 fragment, including exons 12 and 13, description in the main text	Hori et al. (2013)
Becker muscular dystrophy (BMD)	*DMD*	c.3432+1G>A		Exon 25 skipping	Point mutation at the canonical splice site leads to the whole exon skipping	Habara et al. (2009)
Familial dysautonomia (FD)	*ELP1(IKBKAP)*	c.2204+6T>C		Exon 20 skipping (tissue specific)	Shortened transcript has the highest expression in nervous cells, in lymphoid cells, the normal transcript is present at the highest level	Ibrahim et al. (2007), Axelrod et al. (2011)
Type II						
Neurofibromatosis type 1 (NF1)	*NF1*	c.888+651T>A	Deep intronic variants	Cryptic exon inclusion (132 bp)	Mutation creates strong 3′ splice site; this matches with strong, already existing 5′ cryptic splice site. This lead to the inclusion of the cryptic exon containing PTC. Possible admixture of normally spliced transcript	Messiaen and Wimmer (2008)
		c.288+1137C>T		Cryptic exon inclusion (118 bp)	Mutation creates a strong donor splice site which interacts with strong, already existing acceptor cryptic splice site. This lead to the inclusion of the cryptic exon containing PTC	Svaasand et al. (2015)
Cystic fibrosis (CF)	*CFTR*	c.3718-2477C>T		Cryptic exon inclusion (84 bp)	Mutation creates a novel donor site and results in the inclusion of the cryptic exon with PTC	Sanz et al. (2017)
Androgen insensitivity syndrome	*AR*	c.2450-118A>G		Two transcripts: (1) cryptic exon inclusion (85 bp) and (2) cryptic exon inclusion (202 bp)	Mutation creates a novel binding site for splicing enhancer, protein SRSF1 (SF2/ASF)—leading to inclusion of a pseudo-exon containing a PTC	Känsäkoski et al. (2016)
Fabry disease	*GLA*	c. 639+919 G>A		In-frame inclusion of cryptic exon (57 bp)	Mutation abolishes motif that binds splicing silencer protein (hnRNPA1/A2). This leads to the recognition and inclusion of the pseudoexon sequence	Palhais et al. (2016)
Type IV						
Cystic fibrosis (CF)	*CFTR*	c.1525-1G>A	Acceptor splice site mutation	Several transcripts: (1) deletion of exon 10, (2) partial deletion (85 bp) of exon 10, and (3) partial deletion (153 bp) of exon 10	In all three mRNA isoforms, the alternative acceptor sites are used. This lead to the skipping of an exon fragment. Although the bioinformatic analysis showed that the alternative acceptor sites of much greater strength were present in intron 9 than these actually used, there are ESE sites in exons 10 and 11 that drive the splicing process towards the use of particular acceptor sites	Ramalho et al. (2003)
Ehlers-Danlos syndrome	*COL5A1*	c.655-2A>G		Several transcripts: (1) major product exon 5 and 6 skipping, (2) exon 5 skipping, (3) partial deletion (12 bp) of exon 5, and (4) partial deletion (15 bp) of exon 5	The removal of intron 4 is delayed because of the acceptor-site mutation and formation of SECRIs of exons 5 and 6, description in the main text	Takahara et al. (2002)
Duchenne muscular dystrophy (DMD)	*DMD*	c.6614+1G>A	Donor splice site mutation	Partial deletion (32 bp) of exon 45	Mutation at the canonical splice site activates donor cryptic splice site within exon 45 and results in 32 bp deletion at 3′ end of exon 45. Intron 45 has strong acceptor splice site and after its identification, spliceosome is forced to use the nearest donor splicing site. The probability of the use of cryptic donor site is higher	Habara et al. (2009)
X-linked spondyloepiphyseal dysplasia tarda	*TRAPPC2*	c.238+1A>G		Seven different splicing transcripts	Very rare AT splicing donor site is changed into canonical GT splice site that uses major splicing complex instead of minor splicing complex. The activation of cryptic acceptor splice sites is possible and leads to the generation of several alternative transcripts. In this case, bioinformatic software is not effective in identifying the cryptic splice acceptor sites because of the noncanonical ends	Xiong et al. (2009)
Type V						
Medium-chain acyl-CoA dehydrogenase (MCAD) deficiency	*ACADM (MCAD)*	c.362C>T (p.Thr96Ile)	Missense mutation, abolish ESE	Exon 5 skipping	Mutation causes the loss of ESE site and abolishes SF2/ASF protein binding motif thus leading to exon skipping	Ward and Cooper (2010)
Neurofibromatosis type 1	*NF1*	c. 3362A>G (p.Glu1121Gly)	Missense mutation, decreased the ratio of the ESE/ESS	Two transcripts: (1) containing substitution and (2) exon 20 skipping	Mutation results in the presence of two mRNA isoforms: one properly spliced contains missense change (p.Glu1121Gly) and the other one lacks exon 20	Xu et al. (2014)
Stickler syndrome	*COL2A1*	192G>A (p.Cys64Ter)	Nonsense mutation, abolish ESE	Exon 2 skipping	This is an example of class I-NAS. This nonsense mutation (p.Cys64Stop) causes exon 2 skipping by the disruption of ESE. This decreases the level of proper transcript, although shorter mRNA isoforms do not undergo NMD	McAlinden et al. (2008)
Mutations of the branch point sequence						
Neurofibromatosis type 1	*NF1*	2410-18C>G	Branch point	Partial retention (17 bp) of intron 15	Mutation abolishes the original branch point sequence and creates a putative ESE. Other splicing mutations near this position were reported: 2410-16A>G, 2410-15A>G, and 2410-12T>G. It points out that this intron fragment is critical for proper splicing of exons 15 and 16.	Xu et al. (2014)
Xeroderma pigmentosum	*XPC*	c.413-9T>A		Exon 4 skipping	Mutation creates binding site for some factors that prevent the recognition of the weak 3′ splice site at intron 3′ exon 4 junction thus leading to the decrease of normal *XPC* mRNA level	Khan et al. (2010)
	*XPC*	c.413-24A>G		Exon 4 skipping, 3% normal transcript	Mutation abolishes the interaction with U2 snRNP and weak c.413-9 site is used instead of it the normal one resulting in the expression of minimal amounts of full-length *XPC* transcript	Khan et al. (2010)
Mutations within polypirymidine tract						
Hemophilia B	*F9*	c.253-19_253-16del	Polypyrimidine tract	Exon 3 skipping	Mutation shortens the polypyrimidine tract from 24 nucleotides to 20. This results in inefficient splicing and exon 3 skipping	Van de Water et al. (2004)
Mitochondrial acetoacetyl-CoA thiolase (T2) deficiency	*ACAT1*	c.121-13T>A		Exon 3 skipping	Mutation disrupts polypyrimidine tract and causes exon 3 skipping. Moreover, other substitutions in this position (T>A,C,G) also cause exon 3 skipping.	Aoyama et al. (2017)

*PTC* premature termination codon, *NMD* nonsense-mediated decay

The effect of mutation at the canonical splice site might also depend on the strength of the splicing site, localization of cryptic splice sites, density of ESE and ESS, or secondary structures formed by the pre-mRNA. The functional study of selected splicing mutations in the *DMD* gene revealed that the same substitution at the same position from the exon can lead to different effect. The NM_004006.2: c.3277+1G>A mutation in intron 25 leads to the removal of exon 25 from the transcript, while the NM_004006.2: c.6439+1G>A mutation in intron 45 activates the cryptic exonic splicing site resulting in the inclusion of exon 45 shorter by 32 bp at the 3′ end. The experimental studies with minigene assays have confirmed this observation and shown that in the case of the c.6439+1G>A mutation, three distinct splicing isoforms that use different alternative cryptic sites were observed. The authors suggested that the splicing mutation in introns with strong (highly similar to the consensus motifs, e.g., with high complementarity to the 5′ end of the U1 snRNA) splicing motifs, like the donor site of the intron 45 of the *DMD* gene, stimulates the use of cryptic splice sites in exons and introns. The splicing complex specifically recognizes strong splice site, and if the canonical splicing site is mutated, there is a higher probability of the activation of the cryptic splice site. In the case of weak splice sites, the probability of whole exon skipping is higher than the use of alternative splicing motifs. The authors also checked other 14 mutations affecting + 1 position of the *DMD* gene and found that this observation was true for all variants that were localized in exons longer than 170 bp (Habara et al. 2009).

In certain circumstances, several exons might be deleted like in the case of the c.1845+1G>A variant in the *NF1* gene that leads not only to exon 16 but also to the upstream exon 15 removal. During the splicing process of the mutated pre-mRNA, the donor site in the intron 14 and acceptor site in intron 16 are utilized. The proposed model for double exon skipping emphasizes the role of strong consensus splice sites. The exon 15 is surrounded by weak splicing sites, and strong splicing sites of the exon 16 are needed for proper intron 15 and 16 removal. If the strong donor site of the intron 16 is weakened due to the presence of the c.1845+1G>A mutation, the stronger splicing sites at the intron 14 are recognized by the splicing machinery and exon 15 is removed from the transcript (Fang et al. 2001).

The deletion of several exons and generation of distinct mRNA isoforms due to the presence of a splicing mutation might be also related to the order of intron removal. This was analyzed in detail in the case of the c.655-2A>G mutation in the *COL5A1* gene that mutations were found in Ehlers-Danlos syndrome (EDS) patients. In the cells taken from the patient with this mutation, several transcripts were found. The major one was lacking exons 5 and 6, the other one was lacking only exon 5, while in two other transcripts 12 and 15, nucleotides of exon 5 were missing due to the use of the cryptic splicing sites. It was suggested that the major transcript is created because of the delayed removal of the intron 4 due to the presence of acceptor site mutation. As the removal of the intron 5 is not affected, a large multiexon structure called “spliced exon clusters in RNA intermediates” (SECRI) consisting of exons 5 and 6 is formed. Subsequent splicing events can lead to the removal of both exons 5 and 6 and the formation of the major transcript. It can occur, however, that only intron 6 is removed from the SECRI and the transcript that contains spliced exons 5, 6, and 7 can be a substrate for the splicing the used cryptic acceptor sites located in the exon 5. In the case of the transcript lacking only exon 5, it was suggested that the first intron being removed is the intron 6 thus preventing the removal of both exons 5 and 6 (Takahara et al. 2002).

The third mechanism that was proposed to explain the skipping of several exons due to the splicing mutation was the “splicing paralysis” model. It was described in detail for the c.1248+5G>A mutation in *OXCT1* gene that was found in patient with succinyl-CoA:3-ketoacid CoA transferase deficiency (Hori et al. 2013). This mutation leads to the skipping of exons 12 and 13 from the transcript and first studies aimed to identify appropriate SECRI intermediates, but without any success. Further functional analysis had shown that introns in the normal transcript were spliced in the following order: intron 13, 12, and 11. The presence of mutation affecting donor site of the intron 13 caused the delay in this intron removal thus also affecting the time of intron 12 and 11 splicing. Authors suggested that this caused so-called splicing paralysis that could be solved by the removal of whole intron 11-mutated intron 13 fragment, including exons 12 and 13 (Hori et al. 2013).

The effect of a specific splicing mutation may also depend on the tissue type in which the primary transcript is expressed and thus the availability of the specific factors. A good example is the c.2204+6T>C mutation in the *IKBKAP* gene, that is found in homozygous state in about 99% of patients with familial dysautonomia inherited in autosomal recessive manner (Cuajungco et al. 2003; Slaugenhaupt et al. 2001). The bioinformatic analysis with Human Splicing Finder (HSF) did not predict that the c.2204+6T>C variant affects splicing. The functional studies had shown, however, that this substitution caused skipping of the exon 20. Functional analysis of the *IKBKAP* RNA level in distinct tissue types revealed that the wild-type transcript was present at different levels in all examined samples. Its level was the highest in lymphocyte-derived cell lines, while the transcript lacking exon 20 had the highest expression in nervous cells. This can explain why the nervous system deficits are the most prominent in familial dysautonomia patients (Ibrahim et al. 2007; Axelrod et al. 2011).

### Deep intronic variants as a cause of cryptic exon inclusion (type II mutations)

Another category of splicing mutations includes deep intronic mutations—usually substitutions localized within large introns that result in the inclusion of an intron fragment—so-called cryptic exon or pseudoexon, into the mature transcript. Functionally, such variants create novel acceptor or donor sites that are recognized by the splicing complex and are used in combination with the existing intronic cryptic splice sites. It is also possible that deep intronic mutations result in the creation of novel regulatory elements (e.g., splicing enhancers) and the recognition of the specific intronic sequences as an exonic ones (detailed review in Vaz-Drago et al. 2017).

One of the most common and well-known deep intronic change is a c.3718-2477C>T (legacy name: c. 3849+10 kb C>T) variant being one of the most frequent mutations in *CFTR* gene responsible for cystic fibrosis in Polish population (Sobczyńska-Tomaszewska et al. 2013). This mutation is located within the intron 19 and creates a novel donor site that results in the inclusion of an 84-bp cryptic exon into the mature mRNA. This cryptic exon contains an in-frame STOP codon, and thus, the translated protein is shorter and nonfunctional (Sanz et al. 2017). The CF patients with c.3718-2477C>T mutation often have a relatively mild phenotype with a variable disease expression. It was found that the severity of the disease is inversely correlated with the level of correctly spliced transcripts that suggest that the splicing regulation might be an important modifier of the CF clinical course in the presence of intronic mutations. Functional studies had shown that the overexpression of the HTRA2-β1, SC35 splicing factors in the presence of the c.3718-2477C>T mutation promotes proper *CFTR* pre-mRNA splicing and restored the function of the chloride channel (Nissim-Rafinia et al. 2004).

Deep intronic mutations are not common, but their effect on transcript splicing and further protein synthesis is significant. The analysis for the presence of such mutations should be considered when the identification of potentially pathogenic variants in the coding regions and exon/intron boundaries was not effective, and the patient phenotype is specific for a mutation is a specific gene. The techniques that can be helpful in the identification of deep intronic mutations that include RNA/cDNA sequencing and genome or targeted whole gene next-generation sequencing. However, the mutations identified on genomic level should be confirmed with functional RNA testing.

### Exonic mutations affecting splicing (type III and V) mutations

Apart from the intronic mutations affecting canonical splicing sites or activating cryptic exons, also the changes in the exonic sequences may affect the pattern of pre-mRNA splicing. Such exonic mutations might have double effect. First, they can introduce a new 5′ or 3′ splice site or activate the cryptic one that would be stronger than the original one, thus leading to changes in pre-mRNA processing and the loss of an exon fragment (so-called type III splicing mutation). Second, the presence of exonic changes that cause the disruption of exonic splicing enhancers may also lead to the entire exon skipping (so-called type V splicing mutation) (Wimmer et al. 2007).

The application of the RNA/cDNA sequencing in the diagnosis of genetic diseases helps to identify type III and V splicing mutations. In genes that are analyzed with this approach, many different variants were identified and shown to influence proper pre-mRNA processing. According to LOVD mutation database (accessed on 10.10.2017), 26 exonic splicing mutations were identified in the *NF1* gene. Eighteen of them (69%) affected regulatory splicing sequences, especially ESE motifs, resulting in the specific exon skipping. The other mutations (8.31%) created a new cryptic splice site thus leading to the deletion of the exon fragment.

The exonic mutation causing splicing alterations can be easily misclassified as synonymous, missense, or nonsense variant. Usually, the presence of such variants results in the generation of two different transcripts from one mutated allele: one has a proper length and has a modified nucleotide, and the other one is shorter and lack whole exon or its fragment due to the nonspecific activity of the splicing complex (Nissim-Rafinia and Kerem 2002). For example, the presence of c.3362A>G variant in the *NF1* gene results in two mRNA isoforms: one properly spliced contains the substitution that can lead to missense change at the protein level (p.Glu1121Gly), and the other one lacks exon 20. The transcript mosaicism was confirmed with in vitro quantitative studies in two patients harboring this mutation. One of them had higher level of the transcript lacking exon 20, although it did not seem to correlate with the disease severity as both patients presented similar phenotype. It was suggested that the level of different transcript isoforms might be related to the individual genetic variability (Xu et al. 2014).

It is worth to mention in this section the process called nonsense-associated altered splicing (NAS) that was described as a mechanism that should protect transcripts containing premature STOP codons from the nonsense-mediated decay (Cartegni et al. 2002). During this process, the transcripts containing PTC are alternatively spliced and fragments with premature nonsense codon are removed from mature RNA. The exact mechanism of this process has not been described in detail, but it is obvious that in certain circumstances, it is dependent from the frameshift of the reading frame and not from the ESE disruption (so-called class II-NAS). This process was described in the context of the regulation of TCRβ transcripts (Wang et al. 2002). More common are examples of class I-NAS—altered splicing due to the presence of the nonsense mutation that affects ESE site and results in whole exon skipping (Bühler and Mühlemann 2005). Such variants have been described in the *FBN1*, *BRCA1*, or *COL2A1* genes (see Table 1).

### Mutations affecting branch point and polypyrimidine tract

As mentioned, the splicing process is also dependent from the presence of specific sequences: branch site and the polypyrimidine tract sequences that bind specific proteins involved in the formation of splicing complexes. The branch point motif, localized between − 9 and − 400 bp downstream from the acceptor site with the consensus sequence YUNAY in humans, is essential for early spliceosome complex formation. As the sequences of the branch point are highly degenerated, their exact localization is difficult to determine. It seems, however, that mutations localized in the branch point sequence might lead to an exon skipping due to improper binding of the SF1 and U2 snRNP splicing proteins and disruption of the natural acceptor splicing site. Mutations in branch point sequence can also cause intron retention (whole or its fragment) if they create new 3′ splice site (Caminsky et al. 2014).

The polypyrimidine tract with sequence enriched in pyrimidine nucleotides /(Y)_12–17_/ is located between 5 and 40 bp from the acceptor splice site, upstream from the branch point sequence. This sequence binds the U2AF65 spliceosome subunit and polypyrimidine tract-binding protein that is involved in alternative splicing regulation. Any mutations in this sequence probably lead to splicing alterations, although the list of such variants is limited (Ward and Cooper 2010).

Point mutations at the branch point and polypyrimidine tract are very rare and hard to identify when the genomic DNA, mainly coding sequences, are analyzed. As the consensus sequences of these motifs are degenerated, it is hard to predict their exact localization and therefore conclude about the possible effect of a specific variant in these regions. Many of mutations affecting branch point or polypyrimidine tract described so far have been identified by the RNA/cDNA sequencing, or their effect was assessed in functional studies with minigene assay (see “Functional analysis of splicing mutation” section). Such analysis should be performed to confirm the splicing effect of an intronic variant identified near the acceptor splice site (Lewandowska 2013).

## How to detect and confirm splicing mutation?

About 9% of all mutations reported in the Human Gene Mutation Database (HGMD) are splicing mutations (18761/208368) (HGMD database, accessed on October 10, 2017), although it is obvious that this number may be underestimated. Most of the reported mutations were identified by genomic DNA sequencing, and it cannot be ruled out that the number of missense or nonsense substitutions is in fact splicing mutations especially when occur within *cis*-acting elements. Only in several monogenic disorders (e.g., neurofibromatosis type I), the RNA sequencing was implemented into routine molecular diagnosis, mainly due to the problems with RNA stability and availability. Nevertheless, the application of this method allowed to identify synonymous (silent) and nonsynonymous/nonsense mutations as well as deep intronic mutations that affected pre-mRNA splicing. This underlined the fact that the analysis of the DNA sequences (exome or gene) should also include the identification of possible splicing mutations. As the functional testing is challenging, the in silico algorithms were developed to test for possible splicing alterations (Table 2).

**Table 2:** Prediction algorithms for the analysis of splicing effect

Prediction of splice sites		
NetGene2	http://www.cbs.dtu.dk/services/NetGene2/	Hebsgaard et al. (1996), Brunak et al. (1991)
Splice Site Prediction by Neural Network	www.fruitfly.org/seq_tools/splice.html	Reese et al. (1997)
SplicePredictor	http://brendelgroup.org/bioinformatics2go/SplicePredictor.php	Brendel et al. (2004)
Splice port	http://spliceport.cbcb.umd.edu/	Dogan et al. (2007)
SpliceView	http://bioinfo.itb.cnr.it/oriel/splice-view.html	Shapiro and Senapathy (1987), Rogozin and Milanesi (1997)
Analyzer Splice Tool	http://ibis.tau.ac.il/ssat/SpliceSiteFrame.htm	Carmel et al. (2004)
GENSCAN	http://genes.mit.edu/GENSCAN.html	Burge and Karlin (1997)
GeneSplicer	http://www.cbcb.umd.edu/software/GeneSplicer/gene_spl.shtml	Pertea et al. (2001)
MaxEntScan	http://genes.mit.edu/burgelab/maxent/Xmaxentscan_scoreseq.html	Yeo and Burge (2004)
Spliceman	http://fairbrother.biomed.brown.edu/spliceman/	Lim et al. (2011), Lim and Fairbrother (2012)
CRYP-SKIP	http://cryp-skip.img.cas.cz/	Divina et al. (2009)
SROOGLE	http://sroogle.tau.ac.il/	Schwartz et al. (2009)
Human Splicing Finder	www.umd.be/HSF/	Desmet et al. (2009)
MutPredSplice	http://www.mutdb.org/mutpredsplice/submit.htm	Mort et al. (2014)
Alamut Visual Software	http://www.interactive-biosoftware.com	Interactive Biosoftware
MutationForecaster	https://mutationforecaster.com/index.php	CytoGnomix® Inc.
Prediction of branch site and polypyrimidine tract		
Branch Site Analyzer	http://ibis.tau.ac.il/ssat/BranchSite.htm	Kol et al. (2005)
SVM-BPfinder	http://regulatorygenomics.upf.edu/Software/SVM_BP/	Corvelo et al. (2010)
IntSplice	https://www.med.nagoya-u.ac.jp/neurogenetics/IntSplice/	Shibata et al. (2016)
Variant annotations		
Variant Effect Predictor tool	https://www.ensembl.org/info/docs/tools/vep/index.html	Ensembl release 91—December 2017© EMBL-EBI
Alamute Batch software	http://www.interactive-biosoftware.com/alamut-batch/	
Prediction of ESE or ESS		
ESEfinder	http://exon.cshl.org/ESE	Cartegni et al. (2003), Smith et al. (2006)
RESCUE-ESE programs	http://genes.mit.edu/burgelab/rescue-ese/	Fairbrother et al. (2002)
HEXplorer score	https://www2.hhu.de/rna/html/hexplorer_score.php	Erkelenz et al. (2014)
ESRsearch	http://esrsearch.tau.ac.il/	Goren et al. (2006), Fairbrother et al. (2002), Zhang and Chasin (2004)
FAS-ESS	http://genes.mit.edu/fas-ess/	Wang et al. (2004)
SpliceAid2	http://193.206.120.249/splicing_tissue.html	Piva et al. (2012)
SPANR tool	http://tools.genes.toronto.edu/	Xiong et al. (2015)
EX-SKIP	http://ex-skip.img.cas.cz/	Raponi et al. (2011)
HOT-SKIP	http://hot-skip.img.cas.cz/	Raponi et al. (2011)
Prediction of mRNA secondary structure		
mFold	http://unafold.rna.albany.edu/?q=mfold	Zuker (2003)
pFold	http://daimi.au.dk/~compbio/pfold/	Knudsen and Hein (2003)

### In silico analysis of potential slicing mutation

These tools were developed for research purposes, although in certain circumstances can be implemented into routine diagnostics. Algorithms proposed for splicing analysis differ between each other with the database containing information about the consensus sequences, the statistical model used for the analysis, or training methods that are used in machine learning approaches. Most of the tools focus on the analysis of the consensus splicing sites and require the sequence input at least including positions − 3_ + 6 or − 20_ + 3 in the case of 5′ donor and 3′ acceptor sites, respectively. These tools are based on position weight matrix model (Analyzer Splice Tool, SpliceView), probabilistic maximum dependence decomposition model (GENSCAN, GeneSplicer), machine learning techniques (NetGene2, NNSplice—artificial neural networks, SplicePort—support vector machine, SplicePredictor—no web interface, Bayesian model), or maximum entropy distribution model (MaxEntScan). There are also tools developed to detect how distant mutation may influence splicing (Spliceman) or to predict exon skipping, cryptic site activation, or generation of aberrant transcripts from primary sequence (CRYP-SKIP logistic regression model). To predict whether a single nucleotide variant can affect branch site sequence or polypirymidine tract, specific algorithms were developed to identify these sites (e.g., Branch Site Analyzer, SVM-BPfinder) or to predict variant pathogenicity (e.g., IntSplice—support only vcf files; Jian et al. 2014; Ohno et al. 2018).

Also, the effect of a specific variant on the possible ESE or ESS alterations should be examined in the case of exonic mutations. There are several algorithms that can be used to assess these changes like ESE Finder, based on functional SELEX method or RESCUE-ESE Web Server, RESCUE-based MODEL HEXplorer score, and ESRsearch, all three based on relative enhancer and silencer classification by unanimous enrichment approach using frequencies of hexameric sequences. Some models are based on the results of the functional analyses of random sequences for enhancer or silencer properties with minigene assays (FAS-ESS) or the direct interaction between selected splicing factors and their RNA target motifs (SpliceAid2). Only SPANR tool uses splicing code modeling-based approach and was trained on data from different human tissues. The EX-SKIP and HOT-SKIP tools integrate several approaches to analyze potential ESE/ESS sequences (Grodecká et al. 2017). Other bioinformatic programs, such as mFold or pFold, can be used to predict whether a given mutation may affect mRNA secondary structure (Caminsky et al. 2014).

For the maximum comfort of the user, some programs that use different algorithms were developed and made available via the website. The most known are Human Splicing Finder (HSF) and SROOGLE, that predict the presence of cis-splicing elements in the uploaded sequence or generate prediction for a particular variant in a specific gene. Another SNV prediction online tool is MutPredSplice that can analyze single variant or a set of variants uploaded as a vcf file. Multiple algorithms were also implemented in a commercially available software like Alamut Visual Software (Interactive Biosoftware) or MutationForecaster (includes a tool previously known as ASSEDA; CytoGnomix® Inc).

Also, advanced tools used for variant annotations (e.g., from next generation sequencing) can utilize splicing prediction algorithms. For example, the Variant Effect Predictor tool, that is also available online, includes special plugins that perform splicing analysis with MaxEntScan model and dbscSNV matrix (a part of the dbNSFP database). The commercial software for variant annotation, like Alamut Batch software (Interactive Biosoftware), also includes analysis of splicing mutation. The Alamut Batch uses MaxEntScan, HSF, NNSplice, GeneSplicer, and other tools for splicing analysis (user manual available at http://www.interactive-biosoftware.com).

### Functional analysis of splicing mutation

The bioinformatic algorithms are a useful tool in the assessing the possible effect of the identified changes, although it should be underlined that the result of such test is only predictive, and the exact effect of the mutation should be verified in functional studies. An additional test that can be used to confirm that the specific splicing variant has a pathogenic effect is the analysis of a variant segregation with the disease in affected and unaffected family members performed at the DNA level, but still the exact splicing effect should be tested in laboratory (Théry et al. 2011; Fredericks et al. 2015). There are several methods that can be used to analyze the functional effect of a particular variant. The effect of the splicing mutations can be tested on different levels: DNA, RNA, RNA-protein interactions, or protein level itself.

The simplest and the most effective method to determine whether the selected variant affects splicing is to analyze the RNA extracted from a relevant patient tissue or cell line derived from a patient cells. The sequencing of RNA/cDNA after reverse transcription PCR (RT-PCR) allows to verify whether the identified variant influence the mRNA sequence. The main problem with such approach is the possibility of nonsense-mediated decay. In such situation, the effect of the potential splicing mutation can be easily missed out. To overcome this disadvantage, the patient cells can be treated with NMD inhibitors such as puromycin that blocks RNA degradation (Baralle and Baralle 2005). The most common materials used for such functional testing are patient fibroblasts or short-term cultures of blood mononuclear cells (leukocytes).

**Fig. 4:**
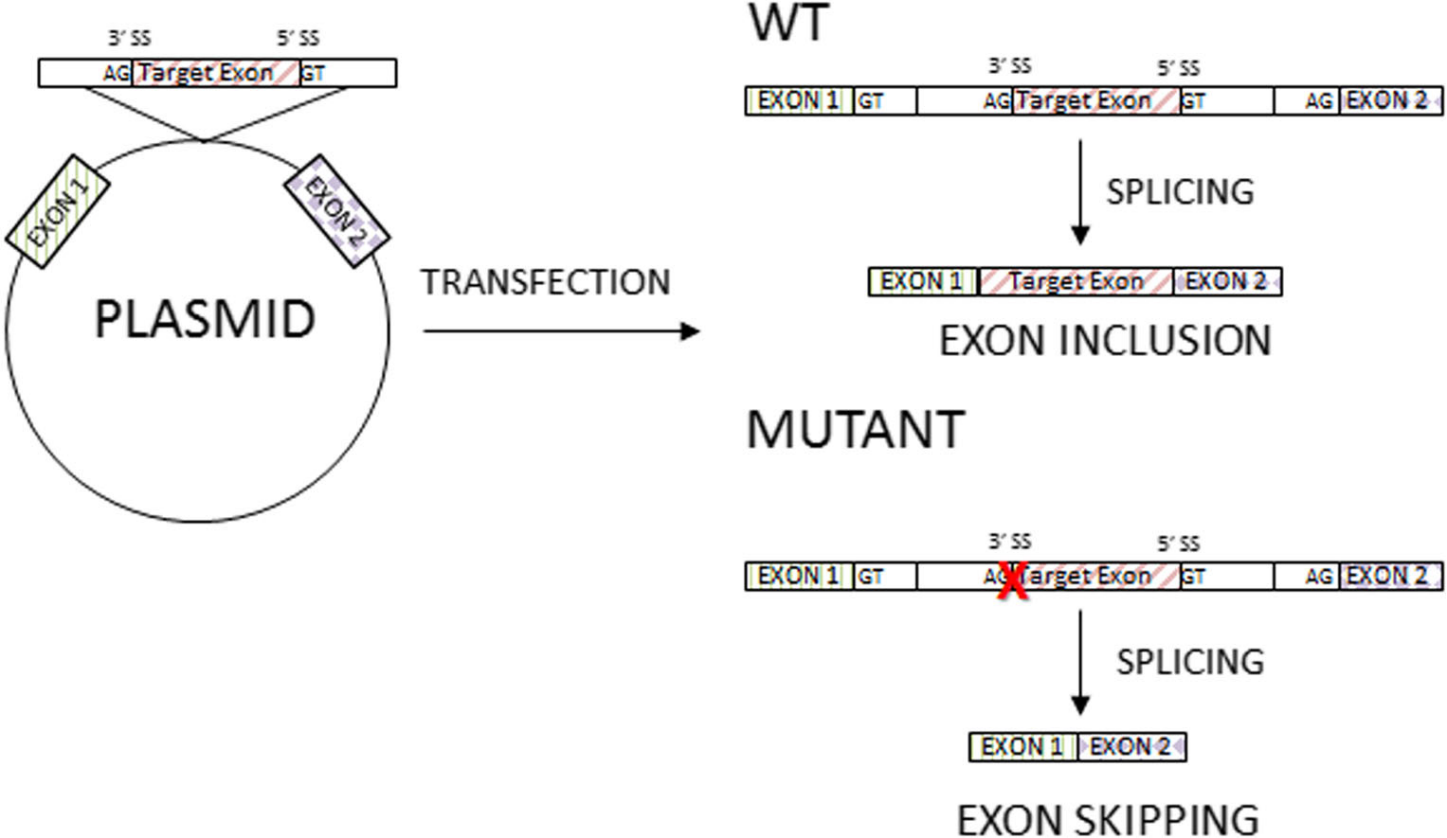
The principles of minigene assay. In the minigene assay, the amplified fragment of the analyzed gene, e.g., specific exon with surrounding intronic sequences with and without mutations, is cloned into a special expression plasmid enabling the analysis the pre-mRNA splicing (see description in text)

If the appropriate material for functional RNA sequencing is not available, an alternative possibility is a minigene assay—an in vitro hybrid system that allows for “exon trapping.” This system is especially useful to the analysis of genes with low expression level in leukocytes or fibroblasts (Singh and Cooper 2006). In minigene assay, the amplified fragment of the analyzed gene, e.g., specific exon with surrounding intronic sequences with and without mutations, is cloned into a special expression plasmid enabling the analysis the pre-mRNA splicing (Fig. 4). This approach can be used to confirm that the potential splicing variant affects splicing efficiency or causes the activation of the alternative cryptic splicing sites, and to test the role of a cis-acting elements on splicing regulation (Sharma et al. 2014).

These techniques are limited because they do not assess the relative level of transcript isoforms. The most valuable method to resolve this problem is real-time PCR (RT-PCR) or quantitative PCR (qPCR) which enables to measure the quantity of each mutated transcript and compare it to the level of the nonmutated one. This method also allows to test whether the mutated transcript undergo NMD (Xu et al. 2014).

The protein truncation test (PTT) was designed as a screening tool to identify mutations that lead to the formation of PTC and resulting in shorter proteins. The test consists of four steps: (1) generation of a template for protein synthesis by amplification of the DNA fragment or RT-PCR of the mRNA, (2) in vitro template transcription and translation in the presence of labeled methionine or leucine amino acids, (3) electrophoresis (SDS-PAGE) of the synthetized proteins, and (4) visualization and assessment of the protein size. When the splicing, nonsense, or frameshift mutation that results in PTC is present, the shorter than expected protein is synthetized. Using this method, the protein abundance and its size are easy to assess (Hauss and Müller 2007). Nowadays, this method was replaced by Sanger or next-generation sequencing, but it remains an option to test for possible splicing defects resulting in shortened protein.

## Summary

It is clear that mutations affecting splicing pattern may be the cause of genetic disorders, although their frequency might be underestimated. During the genomic DNA analysis, they can be easily overlooked and erroneously classified as synonymous changes or benign amino acid substitutions. However, the analysis of RNA/cDNA clearly shows that such mutations have a significant impact on the pre-mRNA splicing. It was assumed that larger genes with long introns were more prone to splicing defects, but it is now more obvious that also a significant number of mutations in smaller genes also cause abnormal mRNA splicing (Chen and Manley 2009). In addition, the analysis of *NF1* and *ATM* genes showed that many of the identified splicing mutations were located outside the canonical splicing sites and could be easily missed during genomic DNA analysis. There is a growing evidence that misclassification of mutation is a common error and the overall number of splicing defects is probably underestimated (Xiong et al. 2015).

The importance of splicing mutations in the pathogenesis of genetic diseases gave rise to numerous experimental and clinical studies that focus on the development of drugs that can reverse the effect of splicing mutations. The most promising approach is the use of antisense oligonucleotides (AONs; Pros et al. 2009; Bergsma et al. 2018). The AONs are short synthetic DNA or RNA molecules that recognize complementary target pre-mRNA fragments and can modulate its splicing by blocking of binding sites for proteins involved in the regulation of the splicing process (Siva et al. 2014). Synthetic AONs can be used to prevent aberrant splicing or to induce additional exon exclusion to restore the proper reading frame. The first approach is used in the therapy of spinal muscular atrophy (SMA), autosomal recessive disorder in 95% of cases caused by homozygous deletion of exon 7 of the *SMN1* gene (Jedrzejowska et al. 2010). In the 5q13.2 *locus*, there is a paralogue of the *SMN1* gene—the *SMN2* gene that differs with five nucleotides from the original one. It encodes the identical protein, although the *SMN2* transcript processing is ineffective due to the presence of c.840C>T substitution in the exon 7. Its presence results in exon 7 skipping in about 80% of *SMN2* transcripts, and a truncated, quickly degraded nonfunctional protein is synthetized. Two models have been proposed to explain exon 7 skipping. First assumes that the presence of c.830C>T substitution causes ESE disruption and inhibits the binding of SF2/ASF splicing activators (Cartegni and Krainer 2002). The second one proposes that the substitution leads to the formation of the exonic splicing silencer that binds hnRNPA1 and represses exon 7 inclusion to the transcript (Kashima et al. 2007; Cartegni et al. 2006). Nevertheless, the Spiranza—an ASO drug that was developed for SMA patients—blocks splicing silencers near the exon 7 of the *SMN2* gene thus generating properly spliced (exon 7 included) transcripts that encode functional SMN protein. The clinical testing has shown high efficacy of Spiranza treatment in patients with all types of SMA (Chiriboga 2017).

The approach that induces exon skipping to restore reading frame was developed for Duchenne muscular dystrophy patients with frameshift mutations in the *DMD* gene. In these patients, it was proved that large in-frame deletions are less deleterious than mutations causing loss of reading frame, and this triggered the idea of the restoration of a reading frame with ASO. First studies focused on the exon 51 skipping, as the deletion of this exon is frequently seen in DMD patients, and the developed drugs (drisapersen and eteplirsen) were successfully used in clinical trials (Veltrop and Aartsma-Rus 2014). The promising results of the ASO-based therapies demonstrate that the treatment of genetic disorders caused by splicing defects is possible and this points out the importance of the identification of such mutations in clinical samples.
